# Identification and partial characterization of new cell density-dependent nucleocytoplasmic shuttling proteins and open chromatin

**DOI:** 10.1038/s41598-023-49100-6

**Published:** 2023-12-08

**Authors:** Kangjing Li, Yaxin Li, Fumihiko Nakamura

**Affiliations:** School of Pharmaceutical Science and Technology, Tianjin University, Nankai District, 92 Weijin Road, Tianjin, 300072 China

**Keywords:** Biochemistry, Cell biology

## Abstract

The contact inhibition of proliferation (CIP) denotes the cell density-dependent inhibition of growth, and the loss of CIP represents a hallmark of cancer. However, the mechanism by which CIP regulates gene expression remains poorly understood. Chromatin is a highly complex structure consisting of DNA, histones, and trans-acting factors (TAFs). The binding of TAF proteins to specific chromosomal loci regulates gene expression. Therefore, profiling chromatin is crucial for gaining insight into the gene expression mechanism of CIP. In this study, using modified proteomics of TAFs bound to DNA, we identified a protein that shuttles between the nucleus and cytosol in a cell density-dependent manner. We identified TIPARP, PTGES3, CBFB, and SMAD4 as cell density-dependent nucleocytoplasmic shuttling proteins. In low-density cells, these proteins predominantly reside in the nucleus; however, upon reaching high density, they relocate to the cytosol. Given their established roles in gene regulation, our findings propose their involvement as CIP-dependent TAFs. We also identified and characterized potential open chromatin regions sensitive to changes in cell density. These findings provide insights into the modulation of chromatin structure by CIP.

## Introduction

CIP regulates gene expression to inhibit cell proliferation when cells reach high density, but the loss of CIP leads to tumor formation^1,2^. Although the nucleocytoplasmic translocation of YAP1, a well-known TAF of the Hippo pathway, in CIP is widely recognized^3,4^, little is known about the gene regulation mechanism in CIP. The aim of this study is to comprehensively determine TAFs and their binding loci on chromatin to uncover the relationship between gene expression and biological phenotypes. Chromatin is composed of DNA spooled around histones in a manner that dynamically exposes specific loci of DNA to TAFs that regulate transcription and replication by binding to the DNA. Systematic profiling of chromatin requires both the detection of the genomic location of exposed loci and the identification of the TAFs bound to each locus. The former is well achieved by current techniques. For instance, histone-3 K4me3 modification is associated with gene expression activity, and a DNA fragment attached to H3K4me3 can be sequenced and mapped on the genome by chromatin immunoprecipitation with sequencing (ChIP-seq) using anti-H3K4me3 antibody and next generation sequencing (NGS)^5,6^. The latter, however, has proved to be more challenging as TAFs are expressed at low levels and require purifying the complex to a high degree without detaching a TAF from a DNA fragment.

To investigate changes in the chromatin profile due to CIP, we stabilized the DNA-TAF complex using dithiobis(succinimidyl propionate) (DSP) and cleaved off the complex using micrococcal nuclease (MNase). We demonstrate that this complex is not only suitable for mass spectrometry analysis of the bound TAF proteins but also for NGS. This DSP-MNase-proteogenomics approach identified many proteins that dissociate from chromosomal DNA when the cells reach at high density and revealed the relationship between their binding loci and biological systems. Specifically, our investigation led to the discovery of TIPARP, PTGES3, CBFB, and SMAD4 as nucleocytoplasmic shuttling proteins that respond to cell density changes, and they are predicted to function as TAFs. Furthermore, we delineated and characterized regions of open chromatin that exhibit sensitivity to cell density alterations. These findings provide fresh insights into the dynamic alterations in chromatin structure orchestrated by CIP.

## Results

### The DSP-MNase-proteogenomics

**Figure 1:**
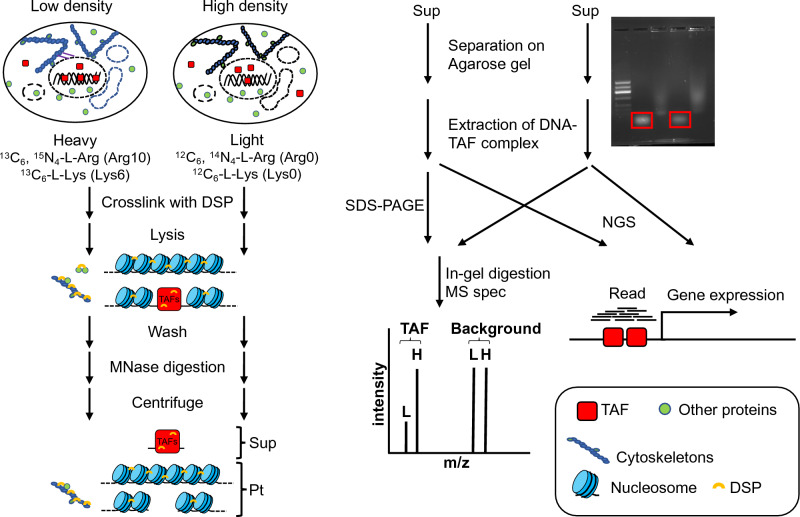
Overview of the DSP-MNase-proteogenomics. Cells were labeled with heavy and light amino acids medium and cultured at low and high density, respectively. DNA–protein complex was stabilized by DSP. After lysing the cells with RIPA-G buffer, soluble materials were washed out and insoluble DNA–protein complexes were digested with micrococcal nuclease (MNase). After centrifugation, soluble DNA-TAF complexes were separated on an agarose gel. The complex was extracted from the gel, de-crosslinked, and subjected to mass spectrometry and next-generation sequencing (NGS). Alternatively, the soluble DNA-TAF complex was de-crosslinked, separated on NuPAGE gel, digested by trypsin in gel, and analyzed by mass spectrometry. Sup: supernatant, Pt: pellet.

To identify TAFs on open chromatin and characterize DNA sequence of open chromatin by NGS, we modified the previously published methods and combined them^7–11^. This method involves the enrichment of a highly concentrated DNA-TAF complex that has been crosslinked by DSP and then cleaved by MNase. The resulting DNA-TAF complex is not only suitable for proteomics analysis but also for genomics. As such, we propose this method be named DSP-MNase-proteogenomics (Fig. 1).

**Figure 2:**
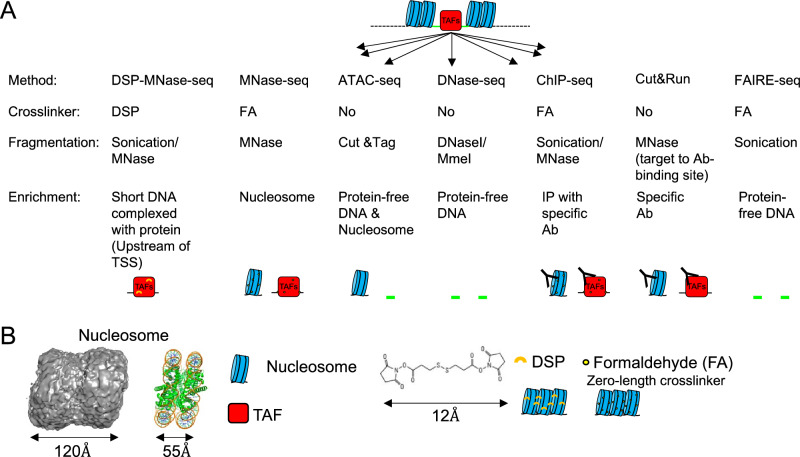
Methods to analyze chromatin structure. (**A**) Comparison of methods for chromatin structure analysis. DSP-MNase-seq specifically enriches the DNA-TAFs complex. Ab: antibody, IP: immunoprecipitation. (**B**) Structure of two nucleosomes (emd_13365) and a single nucleosome (PDB: 7WLR). Note that 12.0 Å-length DSP is a cleavable crosslinker that is sufficiently long enough to crosslink the neighboring nucleosomes, whereas zero-length crosslinker FA may not.

Although YAP1 is a widely recognized TAF that resides in the nucleus of proliferating cells and is relocated to the cytoplasm upon CIP^4,12^, we found that the commonly used nuclear fractionation protocol did not retain YAP1 in the nuclear fraction (Figure S1)^13^. This suggests that previously published methods are insufficient for identifying TAFs that weakly interact with chromatin. This is because these mass spectrometry-based proteomics relies on nuclear fractionation, which may not preserve the DNA-TAF complex during enrichment. To address these limitations, we stabilized the DNA-TAF complex by using a chemical crosslinker. Formaldehyde (FA) is a commonly used chemical to crosslink the DNA–protein complex. However, FA is a zero-length crosslinker that can stabilize DNA–protein and protein–protein interactions only if they are in direct contact, and it may not effectively capture certain DNA–protein complexes^7^. Because neighboring nucleosomes are not cross-linked, each nucleosome is separated after digestion or sonication. Therefore, previously published methods also detect nucleosome DNA or protein-free DNA (Fig. 2A)^10,11,14–16^. To enrich only the DNA-TAF complex, we used DSP because it is long enough to crosslink neighboring nucleosomes, which can be easily removed by centrifugation (Fig. 1 and Fig. 2B) and cross-linking is easily reversed with commonly used reducing agents such as DTT. Although previous research showed that DSP is superior to FA for ChIP-seq analysis of some protein^7^, due to hydrogen bonding in double-stranded DNA, amine in DNA is unreactive towards NHS ester (Figure S2A). Therefore, stabilization of DNA–protein complex is attributed to crosslinking of protein complex wrapped around DNA rather than direct crosslinking between protein and DNA. Therefore, we tested if DSP can stabilize YAP on DNA and the YAP-DNA complex can be separated from nucleosome.

**Figure 3:**
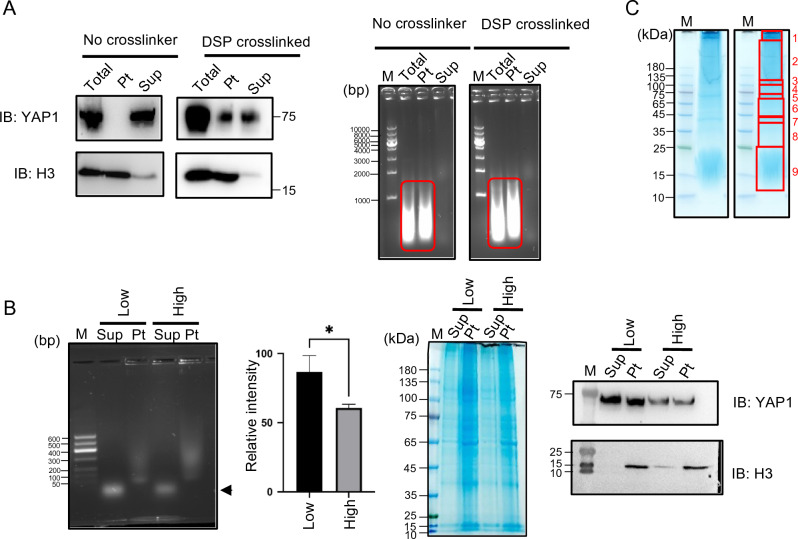
Preparation of DNA-TAF complex. (**A**) DSP-mediated cross-linking maintains the YAP-DNA complex. Human skeletal muscle (hsSKM) cells were treated with or without DSP, subsequently quenched, and lysed using RIPA buffer. The lysate underwent centrifugation to differentiate between the soluble (supernatant, Sup) and insoluble (pellet, Pt) fractions. Each sample was then analyzed by western blotting to detect YAP1 and histone H3 (left panel), as well as agarose gel electrophoresis to detect DNA (right panel). (**B**) MNase digestion of the DSP-crosslinked chromatin. Crosslinked nucleosomes (containing histones) are fractionated in the pellet after MNase digestion and centrifugation. Digested small DNA fragments (arrow) are fractionated in the supernatant that also contains proteins including YAP1 (left panel). The bar graph shows the relative intensity of the DNA bands in the supernatant, indicating that more DNA–protein complex was detected from low-density cells than from high-density cells (second panel from the left). SDS-PAGE gel stained with CBB for each fraction corresponding to the left panel. (third panel from the left). Consistent with the localization of YAP1, more YAP1 was extracted from low-density cells than from high-density cells (right panel). IB: immunoblotted by anti-YAP antibody and anti-histone H3 antibody. (**C**) CBB stained SDS-PAGE gel for SILAC-based proteomics. Proteins bands were cut into 9 fragments and subjected to in-gel trypsin digestion for mass spectrometry. Sup: supernatant, Pt: pellet.

To enrich the YAP-DNA complex, the DSP crosslinked cells were lysed with RIPA-G buffer. Addition of 6 M guanidine in the lysis buffer effectively removed the majority of proteins, including actin, while retaining YAP1 in the complex (Figure S2B). Without crosslinking, YAP1 was dissociated from DNA during washing (Fig. 3A).

Consistent with the subcellular localization of YAP1, more YAP1 was detected in the enriched DNA–protein complex from the low-density cells compared to that from the high-density cells (Fig. 3B left panel). Additionally, more DNA was also observed in the enriched DNA–protein complex from low-density cells than that from the high-density cells, suggesting that chromatin is more open in low-density cells than in high-density cells (as indicated by the arrow and bar head in Fig. 3B). Furthermore, even following rigorous washing, a substantial amount of protein suitable for proteomic analysis remained associated with the DNA (Figure S2B). Currently, we do not have a complete understanding of why approximately half of YAP1 was located in the pellet fraction after MNase digestion (Fig. 3B, right panel). Nevertheless, our primary focus was on identifying TAFs that bind to open chromatin, and the investigation of YAP1's characteristics within the pellet was beyond the scope of this study. To identify proteins bound to DNA in a cell density-dependent manner, we employed two different proteomic approaches. First, we simply extracted protein in the band on agarose gel (Fig. 3B, arrow head) followed by conventional mass spectrometry. Another approach was to use stable isotope labeling using amino acids in cell culture (SILAC) to detect proteins specifically enriched in the MNase-digested fraction from low-density cells or high-density cells (as shown in Fig. 1 and 3C).

### The purified DNA–protein complex is amenable for proteomics

**Figure 4:**
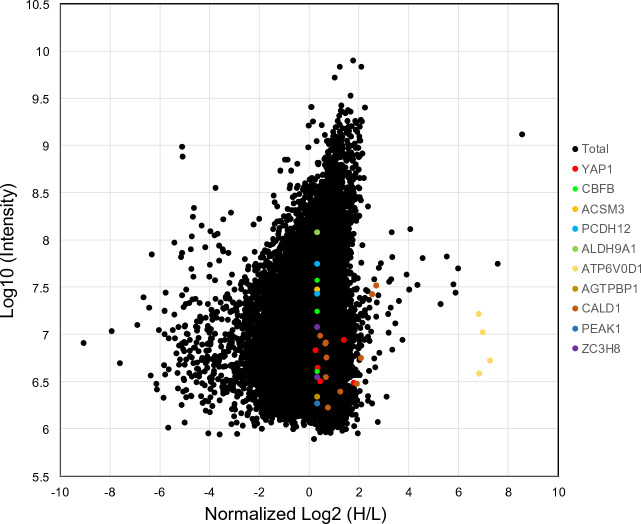
Analysis of the SILAC-based mass spectrometry of purified DNA protein complex. Standard scatterplots with normalized Log_2_ (H/L) ratios/Log_10_ intensities highlight the distribution of quantified peptides in each MS screening.

Mass spectrometry of peptides from the extracted DNA-proteins complex identified 255 proteins (Table S1 and S2, column A). Among them, 52 proteins were predicted to be related to transcription after Uniprot database search (Table S2, column B). We also analyzed proteins bound to the MNase-extracted DNA using SILAC-based proteomics (Fig. 1). This analysis detected 3647 different proteins but peptides from 1220 different proteins were exclusively detected on the right side (positive Log(H/L)) of the scatterplot (Tables S3 and S4, Fig. 4), indicating that these 1220 proteins were enriched in low density cells. Even though the normalized H/L ratio for some of the identified peptides resulted in NaN (not a number), we chose to include these candidates, such as SMAD4 in Table S5. We made this decision because NaN would imply an exceptionally high H/L ratio. Among the 1220 detected proteins, 179 were predicted to be associated with transcription following a search in the Uniprot database (Table S4, column B).

However, these proteomics detected seemingly irrelevant proteins such as cytoskeletal caldesmon 1 (CALD1) and mitochondrial acyl-coenzyme A synthetase (ACSM3) (Fig. 4), indicating the need for further screening. Since YAP1 is known to translocate between the nucleus and cytosol in a cell density-dependent manner, we searched for localization of the candidate proteins (52 candidate proteins from the conventional mass spectrometry as shown in Table S2 column B and 179 candidate proteins from the SILAC-based mass spectrometry as shown in Table S4 column B) using the subcellular map of the human proteome on the Human Protein Atlas database (https://www.proteinatlas.org). From the candidate proteins that we detected by the two different proteomics, we further screened a protein that is localized in the nucleus at low density and in the cytosol at high density. If this information was not available, we selected a protein that is differently stained in the nucleus and cytosol in different cell types on the database. Because in this database even YAP1 is stained in the nucleus only or both in the nucleus and cytosol depending on cell types. A protein that is stained only in the nucleus or cytosol was eliminated. This screening narrowed down our list of candidates to 17 proteins that may translocate between the nucleus and cytosol upon CIP (Table S5).

### TIPARP, PTGES3, CBFB, and SMAD4 are novel CIP-sensitive TAFs

**Figure 5:**
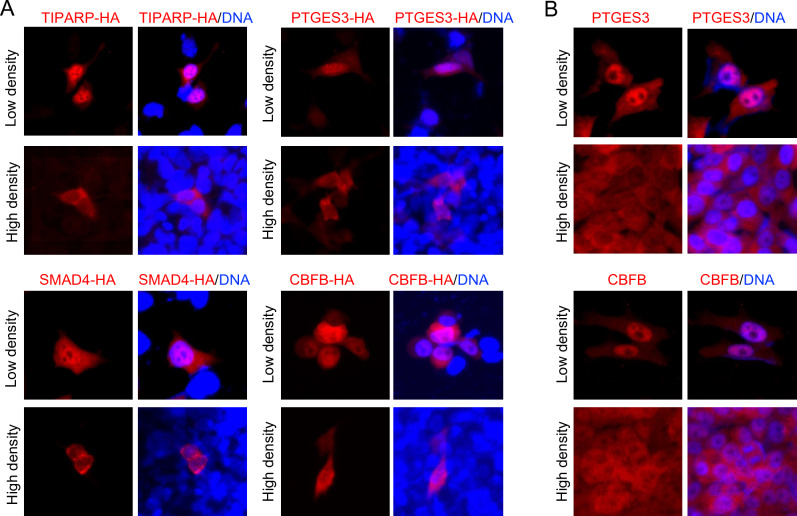
Cell density-dependent translocation of TIPARP, PTGES3, SMAD4, and CBFB. (**A**) HEK 293 cells were cultured at either low or high density and transfected with either pcDNA3-TIPARP-HA, pcDNA3-PTGES3-HA, or pcDNA3-SMAD4-HA. For CBFB, hsSKM cells were transfected with pcDNA3-CBFB-HA. Expressed proteins were detected by immunofluorescent microscopy using an anti-HA antibody followed by a secondary antibody conjugated with Alexa594. (**B**) HEK 293 cells were cultured at either low or high density for the detection of PTGES3, and hsSKM cells were cultured at either low or high density for the detection of CBFB. Both cell types were then fixed. PTGES3 was detected using immunofluorescent microscopy with a specific anti-PTGES3 antibody, followed by a secondary antibody conjugated with Alexa594. CBFB was detected using immunofluorescence with a specific anti-CBFB antibody, followed by a secondary antibody conjugated with Alexa594. Nuclei were stained with Hoechst 33,342. Scale: 100 ✕ 100 μm.

To determine if these candidate proteins truly respond to changes in cell density, we expressed them exogenously with a hemagglutinin (HA) tag at their C-terminus using a mammalian expression vector and their localization by immunofluorescent microscopy (IF). Our results showed that TIPARP, PTGES3, CBFB, and SMAD4 are primarily located in the nucleus of low-density cells, but they translocate to the cytosol when cells reach high density (Fig. 5A and Figure S3). Consistent with this observation and the SILAC proteomics data, a higher amount of CBFB was fractionated in the MNase-extract from low-density cells compared to high-density cells. (Figure S3D). Since overexpression of the exogenous protein might impact on the subcellular localization, we reevaluated the result using commercially available specific antibodies. Direct staining of PTGES3 and CBFB in HEK 293A cells at low and high densities further confirmed that their localization is dependent on cell density (Fig. 5B and Figure S3). We tested if DSP could replace commonly used FA for IF. Staining of CBFB in low- and high-density cells fixed with DSP was consistent with those fixed with FA (Figure S4), demonstrating that DSP does not modify the epitope of the anti-CBFB antibodies we used.

### Computational analysis of biological roles of TIPARP, PTGES3, and CBFB in CIP

**Figure 6:**
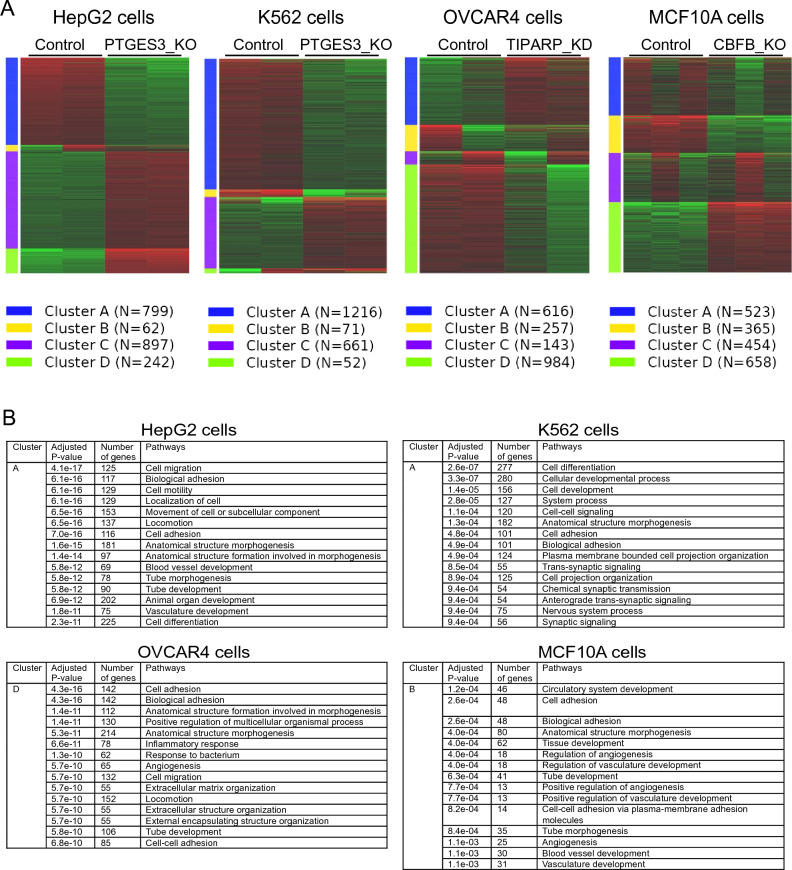
RNA-seq analysis demonstrated that all identified CIP-sensitive molecules are involved in cell migration, adhesion, and morphogenesis. (**A**) PTGES3 knock-out (KO) in human hepatoma HepG2 cells (BioProject: PRJNA30709). (**B**) PTGES3 KO in human immortalized myelogenous leukemic K562 cells (BioProject: PRJNA30709), (**C**) TIPARP/PARP7 knock-down (KD) in human ovarian carcinoma OVCAR4 cells (BioProject: PRJNA642215), (**D**) CBFB KO in human breast epithelial MCF10A cells (BioProject: PRJNA492137).

To investigate the biological role of the identified cell density-sensitive nucleocytoplasmic shuttling molecules, we searched public literature and databases. We analyzed publicly available RNA-seq data of cells depleted of TIPARP, PTGES3, and CBFB to decipher their biological features. The RNA-seq analysis revealed that these genes are associated with cell migration, adhesion, and development (Tables S6, S7, S8, S9 and Fig. 6). These data suggest that the identified proteins are suppressed in CIP by being translocated from the nucleus to cytosol to down-regulate cell migration and adhesion.

### The purified DNA–protein complex is amenable for genomics

**Figure 7:**
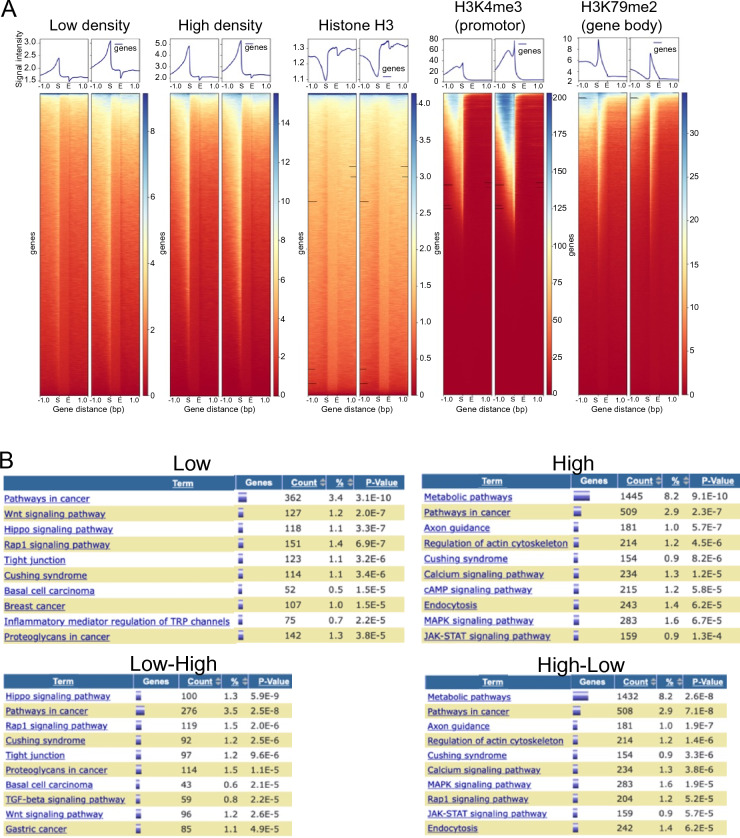
Purified DNA protein complex is amenable for genomics of open chromatin. (**A**) The top section displays the profiles, while the bottom section shows the heatmaps of MNase-digested DNA fragments derived from hsSKM cells with varying densities (low and high). We acquired ChIP-seq data for histone-H3 (analyzed in our laboratory using hsSKM cells), H3K4me3, and H3K79me2 in human skeletal muscle cells from a publicly accessible database. TSS and transcription end site (TES) are denoted by S and E, respectively. The gene body is situated between the TSS and TES. Notably, the MNase-digested DNA fragments from low- and high-density cells exhibit enrichment upstream of the TSS. Two representatives are independently shown. (**B**) We combined the two representatives and conducted KEGG pathway analysis on genes linked to genomic regions identified through NGS of MNase-digested DNA fragments from both low- and high-density cells. Utilizing NGS data, we employed GREAT (http://great.stanford.edu/public/html/) to analyze the genes associated with these genomic regions. Subsequently, we assessed the identified genes' functional annotations in KEGG using DAVID (https://david.ncifcrf.gov/tools.jsp). Notably, the genes linked to genomic features in low-density cells, which exhibit no overlap with high-density cells, show significant enrichment in the Hippo signaling pathway. Conversely, the genes associated with genomic features in high-density cells, without overlap in low-density cells, are significantly enriched in the metabolic pathway. These findings imply that genes related to the Hippo pathway are downregulated in high-density cells.

To investigate where the MNase-digested DNA fragments bound to TAFs are located in the human genome, we performed an NGS analysis of the purified MNase-digested DNA fragment. To facilitate comparisons, publicly available ChIP-seq data for histone H3, H3K4me3 (a marker for active promoters), and H3K79me2 (a marker for gene bodies) were employed. These markers were selected due to their well-characterized profiles, which are consistent and not influenced by cell type. Although MNase-digested DNA fragments migrated on the agarose gel as ~ 50 bp (Fig. 3B arrow), the sizes of the sequenced DNAs were larger than 150 bp (maximum read length, Figure S5), presumably because DNA covalently cross-linked to TAFs migrated faster on the agarose gel. MNase-digested DNA fragments from both low- and high-density cells are enriched in upstream of transcription start site (TSS), which is also seen in H3K4me3 ChIP-seq data, but not in histone H3 and H3K79me2 ChIP-seq data (Fig. 7A top). The profiles of MNase-digested DNA fragments are notably distinct from those obtained through alternative analyses like MNase-seq and ATAC-seq.^9,17^ as expected (illustrated in Fig. 2A). Given the similar nature of the independently acquired NGS data (Fig. 7A top and Figure S6), these two datasets were combined for subsequent analysis. The profile analysis revealed enrichment of MNase-digested DNA fragments upstream of the transcription start site (TSS), with peaks also present in the gene body (Figure S6). This is likely due to the binding of the elongation complex to the gene body during transcription elongation. The inconsistency in peak locations within this region can be attributed to the use of pooled heterogeneous cells for the analysis. The development of single-cell analysis would offer a solution to this issue.

Next, we used the Genomic Regions Enrichment of Annotations Tool (GREAT)^18^ to analyze the functional significance of the sequence of DNA fragments isolated from low- and high-density cells. GREAT interprets the biological significance of a group of non-coding genomic regions by examining the annotations of genes in proximity. Consequently, it proves especially valuable in investigating the cis functions of sets of non-coding genomic regions. The majority of the peaks in histone H3 ChIP-seq were mapped to the intergenic non-protein-coding region and were not associated with the protein-coding gene (Figure S7). However, similar to H3K4me3 ChIP-seq, about 13% of MNase-digested DNA fragments from both low (12.6%) and high (13.2%) density cells were mapped to promoter region (H3K4me3: 14.3%) and were associated with more than one gene. Despite a lower amount of DNA from the high-density cells than that from the low-density cells (Fig. 3B arrow head), more peaks were detected in the high-density sample (Low: High = 20,787: 159,498) presumably because more specific genomic regions are open in low-density cells than high-density cells and PCR amplification for NGS sequencing enriched such specific regions in low-density cells. These results demonstrated that our DSP-MNase-proteogenomics method can also determine DNA sequence where TAFs bind to regulate gene expression. These results suggest that DNA fragments enriched by our method detect cis-regulatory regions where TAFs bind.

### Genotype–phenotype relationship in CIP

To investigate if sequenced open chromatin is associated with CIP phenotype, we performed functional annotation of detected genes. First, we screened for genomic features of a “Low” density sample that have no overlap in the “High” density one. Genes associated with the genomic region were detected on GREAT (Tables S10, S11) and analyzed on the functional annotation tool, DAVID^19^. KEGG pathway analysis revealed that the “Hippo signaling pathway” is the significant pathway in low-density cells, whereas the “metabolic pathway” is more significant in high-density cells (Fig. 7B). This result is consistent with our current understanding, in which CIP-dependent YAP1 translocation regulates the Hippo pathway to control cell growth^12,20^.

### DSP-stabilized DNA-TAF complex is amenable for ChIP-seq analysis

**Figure 8:**
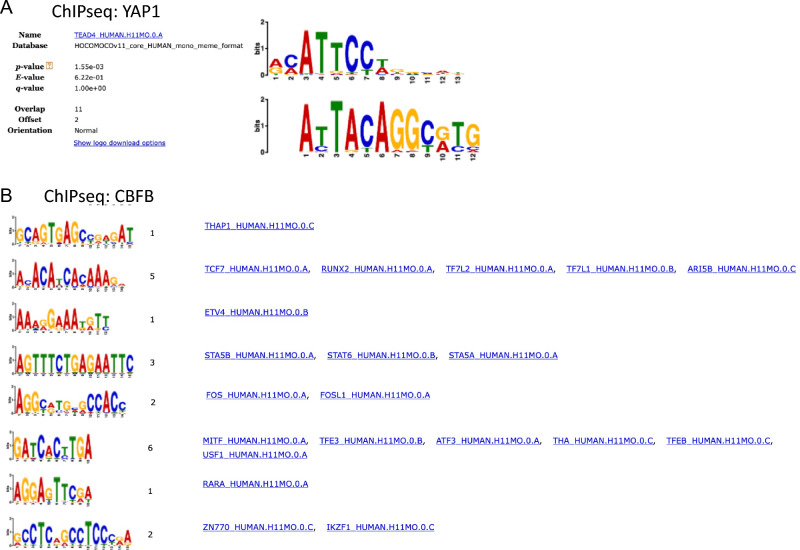
ChIP-seq analysis of DNA fragments bound to YAP1 and CBFB crosslinked by DSP in hsSKM cells. (**A**) Motif analysis for YAP1-binding sites in hsSKM cells detected a known TEAD4-binding motif. (**B**) Motif analysis for CBFB-binding sites in hsSKM cells detected not only known RUNX2-binding motifs but also other transcription factor-binding motifs.

We investigated if the extracted DNA-TAF complex stabilized by DSP can be immunoprecipitated by anti-CBFB antibodies and bound DNA can be sequenced by NGS to identify CBFB-binding motifs. As a control, we used anti-YAP1 antibodies. The YAP1 ChIP-seq identified a known TEAD-binding motif (Fig. 8A) and CBFB ChIP-seq detected not only a known RUNX2-binding motif but also other transcription factor-binding motifs (Fig. 8B)^21–24^. While a direct comparison between conventional ChIP-seq using FA-fixed samples and ChIP-seq with DSP-stabilized DNA-TAF complex is needed, the findings indicate that the DSP-stabilized DNA-TAF complex shows potential to supplement the conventional ChIP-seq analysis for molecules that cannot be immunoprecipitated after FA-fixation.

## Discussion

Currently available mass spectrometry (MS)-based approaches to identify TAF proteins use purification of the nucleus or chromatin^25,26^. However, purifying the nucleus, where chromatin resides, is dubious. The outer membrane of the nuclear envelope is continuous with the membrane of the rough endoplasmic reticulum, thereby making it impossible to fractionate the “pure” nucleus although the so-called “nuclear fractionation protocol” has been widely used. In addition, we found that the commonly used nuclear fractionation protocol does not retain YAP1 in the nuclear fraction. In fact, YAP1 has never been quantitatively detected by MS after fractionation of the nucleus or chromatin without using a crosslinker^25–28^. To overcome these issues, we preserved the DNA-TAF complex for proteomics and genomics studies by using the cell-permeable DSP cross-linker. DSP is useful not only because it is reversible but also because it can cross-link neighboring nucleosomes. In addition, the DSP-stabilized DNA–protein complex can withstand harsh washing with SDS and guanidine, which can remove the majority of cytosolic proteins. Since DSP does not cross-link DNA, this stabilization of DNA–protein complex is mostly like due to stabilization of protein complex that is mechanically and chemically but not covalently wrapped around DNA. Hence, DSP-MNase-proteogenomics can serve as a supplementary method to enhance the existing approaches for profiling chromatin.

Reversibility is essential for downstream mass-spectrometry analysis. Although MaxQuant has an option to consider DSP modification in peptide analysis, we did not see any differences in the detected peptides, presumably because the cross-linking is partial. Cross-linking neighboring nucleosomes is also crucial for enriching the DNA-TAF complex. After extracting open chromatin using MNase digestion, the remaining closed chromatin can be easily removed by a conventional tabletop centrifuge because the cross-linked nucleosomes are large. This is supported by our results, which show that histones were only detected in the pellet after centrifugation, while YAP1 was detected in the soluble supernatant fraction. However, approximately half of YAP1 was still detected in the pellet presumably because some YAP1 molecules are free from open chromatin and instead form complexes with other proteins that can be sedimented in the pellet following centrifugation. Despite this, our method is sufficient for preserving the DNA-TAF complexes, including YAP1. In fact, we have successfully detected many new potential TAFs, and four of them have experimentally been confirmed as cell density-sensitive TAFs. Furthermore, we have also demonstrated that the isolated DNA fragments from the DNA-TAF complex, obtained through proteinase K digestion and decrosslinking to remove proteins, are suitable for NGS analysis. Our method was sufficient to detect DNA sequences on open chromatin, which are associated with cellular phenotypes determined by computational analysis.

FA is commonly used to preserve DNA–protein complexes for chromatin analysis, such as ChIP-seq and FAIRE-seq^29^. The methylene bridge formed by FA is stable but believed to be reversed by heating^30^, enabling downstream sequencing analysis. However, unlike DSP-crosslinked chromatin, FA-crosslinked chromatin requires additional steps for fragmentation by sonication and/or nuclease digestion, and enrichment of the DNA-TAF complex using an antibody targeting a specific TAF. In contrast, the DSP-MNase proteogenomics method enables a thorough examination of all MNase-sensitive DNA sequences (cis-regulatory elements) bound to TAFs, along with the identification of TAFs on open chromatin. Therefore, this approach facilitates a systematic analysis of chromatin. Despite systematic analysis revealing that open regions of chromatin are mainly associated with genes involved in the Hippo pathway in proliferating low-density cells and genes involved in metabolism in high-density cells that have stopped proliferating and migrating, questions remain about how these genes regulate these phenotypes.

The DSP-MNase proteogenomics detected many proteins that associate with or dissociate from chromatin upon CIP. Although we focused on molecules that translocate between the nucleus and cytoplasm in a cell density-dependent manner for ease of screening, it's worth noting that a significant portion of the screened molecules is exclusively localized in the nucleus, as per the data from the Human Protein Atlas database. This suggests that these molecules associate with or dissociate from chromatin in a cell density-dependent manner within the nucleus. Further analysis is necessary to characterize these molecules.

We experimentally showed that among many potential CIP-sensitive TAFs, TIPARP, PTGES3, CBFB, and SMAD4 translocate between the nucleus and cytoplasm in the same fashion as YAP1, depending on cell density. TIPARP (a.k.a PARP7), TCDD-inducible poly [ADP-ribose] polymerase, is an ADP-ribosyltransferase that mediates the mono-ADP-ribosylation of target proteins to control gene expression. For example, it functions as a negative regulator of AHR, a ligand-activated transcription factor, to regulate biological processes such as angiogenesis, hematopoiesis, cell motility, and drug and lipid metabolism ^31,32^. Although AHR disrupts the contact inhibition^33^, the function and mechanisms of TIPARP in the nucleus and cytoplasm are still not fully understood. PTGES3 (also known as mPGES-1), Prostaglandin E synthase 3, also plays a role in the regulation of gene expression through its ability to produce prostaglandin E2. This can activate various signaling pathways and modulate the activity of transcription factors^34^. CBFB, the core-binding factor subunit beta, forms a complex with RUNX family proteins, modulating the transcription of target genes involved in the development of various tissues^23^. SMAD4 (a.k.a DPC4), Suppressor of Mothers Against Decapentaplegic Homolog 4, also regulates gene expression by promoting the binding of multimeric complex to DNA^35^. Therefore, all these CIP-sensitive proteins act as TAFs. However, our findings raise new questions: How does CIP regulate the nucleocytoplasmic translocation of these molecules, or vise vasa? What are their functions in the nucleus and cytoplasm? Does disruption of their translocation correlate with tumorigenesis?

There may be an overlap between the signaling pathways of CIP and mechanotransduction. In fact, the translocation of YAP1 is regulated by both mechanical force and CIP^3^. The cell–cell contact in high-density cells might indirectly regulate YAP1 signaling through Merlin/NF2^36^ or restrict YAP1 nuclear entry by mechanically modulating the size of the nuclear pore through the LINC complex^37^. Given that the sizes of TIPARP (657 amino acids), PTGES3 (160 amino acids), CBFB (182 amino acids), and SMAD4 (552 amino acids) are similar to or smaller than YAP1 (504 amino acids), the stretching of the nuclear pore induced by force might regulate their entry into the nucleus, although biochemical regulation cannot be ruled out.

The functional annotation analysis uncovered a reduction in genes associated with the Hippo signaling pathway in high-density cells. This outcome aligns with our recent findings, which demonstrated that UBE2A/B localizes to the nucleus of low-density cells and induces the ubiquitination of histone H2B^13^. Given that the knockdown of UBE2A/B leads to a decrease in YAP1 expression and UBE2A/B translocates to the cytoplasm in high-density cells, it is plausible to attribute, at least in part, the reduction in gene expression related to the Hippo signaling pathway in high-density cells to the UBE2A/B pathway.

Histone H3, in conjunction with histone H2s and H4, forms the nucleosome. Approximately 146 base pairs of DNA are tightly wrapped around the histone octamer, rendering it generally inaccessible to TAFs (Fig. 2A)^38,39^. Since the signal intensity of ChIP-seq profiles for histone H3 is low in the upstream of the TSS and TAFs are known to bind histone-free regions, it is predicted that the upstream of the TSS serves as the TAF-binding site (cis-regulatory element). Furthermore, the H3K4me3 modification is recognized for its role in recruiting TAFs, and it is associated with both active and poised genomic loci^40–44^. Consequently, the H3K4me3 modification is enriched in the upstream of the TSS. On the other hand, the H3K79me2 modification is enriched within the gene body, between the TSS and TES. Although the template looping model^45,46^ suggests the recycling of these modified histones during elongation, the precise mechanism governing how TAFs bind to chromatin remains enigmatic^47,48^. In theory, DNA should be devoid of histones to permit TAF binding. However, a significant number of epigenetic markers consist of histone modifications, implying that the loci where TAFs bind cannot be discerned by these markers unless modified histones remain in proximity to TAF-binding sites. While further characterization is warranted, the DSP-MNase proteogenomics approach inherently identifies DNA regions bound by TAFs with a few or no histone, setting it apart from existing methods.

In summary, using modified proteomics of TAFs bound to DNA, we identified a protein that shuttles between the nucleus and cytosol in a cell density-dependent manner and comprehensively determined their binding loci on chromatin. These findings shed light on chromatin structure that is altered by CIP. However, additional biological characterization is essential to confirm the involvement of the identified TAFs in CIP.

## Materials and methods

### Cell lines and SILAC labeling

Human skeletal muscle (hsSKM, ATCC) cells were grown for at least six generations in Dulbecco's modified Eagle's medium (DMEM) for SILAC (Thermo) supplemented with L-lysine and L-arginine (light) or L-lysine-^13^C_6_ and L-arginine-^13^C_6_, ^15^N_4_ (heavy) (Thermo) as previously described^49^. 50 mg of each amino acid was added into every 500 mL DMEM for SILAC. HEK293A cells were purchased from Thermo Fisher. These cells were grown in DMEM (Biological Industries, Israel) supplemented with 10% FBS (Biological Industries, Israel) and 1% penicillin–streptomycin. Cells were maintained at 37 °C and 5% CO_2_.

### Purification of DNA–protein complex

RIPA-T buffer: 50 mM Tris–HCl (CotyBioTech), pH 8.0, 150 mM sodium chloride, 1.0% tween-20 (ChemCruz), 0.5% sodium deoxycholate (bidepharm.com), 0.1% SDS (ChemCruz). RIPA-G buffer: 50 mM Tris–HCl, pH 8.0, 150 mM sodium chloride, 1.0% tween-20, 0.5% sodium deoxycholate, 0.1% SDS, 6M guanidine hydrochloride (Solarbio).

Growth medium was replaced with 6 ml of PBS and cells were cross-linked with 1.0 mM dithiobis(succinimidyl propionate) (DSP) (Meilunbio) in 6 ml PBS at room temperature for 30 min. Unreacted DSP was quenched by adding 0.18 ml of 1 M Tris–HCl pH = 8.0 at room temperature for 15 min and the cells were rinsed with PBS 3 times. Fixed cells were lysed with 400 μL RIPA-G buffer + protease inhibitor cocktail (PIC) + RNase to extract DNA–protein complexes. The extract was centrifuged at 15,000 × g for 10 min at 4 °C. The pellet was rinsed with 400 μL PBS 5 times to remove the lysis buffer and then suspended in 400 μL PBS by pipetting. After centrifugation at 10,000 × g for 2 min at 4 °C, the pellet was redissolved in 100 μL RIPA-T buffer + RNase by sonication (1s/1s, 5s). The redissolved DNA–protein complex was digested with 0.5 μL Micrococcal Nuclease (Cell Signaling Technologies) and 0.2μL 1M CaCl_2_ at 37 °C for 30 min. After centrifugation at 20,000 × g for 30 min at 4 °C, the supernatant was collected in a new tube as purified DNA–protein complex.

### Trypsin digestion and mass spec analysis of protein bound to DNA

The collected supernatant was run on 1% agarose gel and DNA was stained with ethidium bromide. The DNA–protein complex (apparent size is around 50 bp) was extracted by glass wool-centrifuge system^50^. The eluate was concentrated to 100 μL. The DNA-complex was precipitated using 0.7 volume of isopropanol and 0.1 volume of 5M NaCl at -20 °C overnight. The pellet was washed by 70% ethanol and redissolved in 100 μL 50 mM ammonium bicarbonate solution. After adding 10 mM dithiothreitol (DTT), the solution was incubated at 60 °C for 30 min. Then 40 mM iodoacetamide (IAA) was added in the mixture and incubated in dark for 30 min. To stop the reaction, 40 mM DTT was added and incubated for another 40 min. The mixture was transferred to a 10 kDa cut-off ultra mini filter unit (Amicon). Buffer was replaced with 50mM ammonium bicarbonate (each 200 μL) 3 times and the solution was concentrated to 75 μL in the filter unit. To digest protein 2.5 μL (100ng/μL) trypsin was added and incubated at 37 °C overnight (12–16 h). The digested peptides were analyzed by LC–MS/MS on Orbitrap Fusion Lumos mass spectrometer (Thermo Scientific, San Jose, CA).

### In-gel trypsin digestion and mass spec analysis of SILAC sample

After DNA–protein complex purification, labeled samples (heavy amino acids for low density cells and light amino acids for high density cells) were mixed in 4 mL Amicon Ultra centrifugal filter unit (3 kDa cut-off). The mixture was centrifuged at 4 ℃ at 13,000 × g until the volume reached to 30μL. Sample was transferred to a new tube and mixed with 10 μL 4 × LDS sample buffer. The sample was heated at 70 °C for 10 min. The protein sample (40 μL) was separated on the SurePAGE (4–12% gradient gel, Genscript) and stained with Coomassie Blue G-250 staining buffer (GenStar) for 2 h and destained with ultrapure water for at least 1 h. The protein bands were cut into 9 slices (Fig. 3C), all of which were subsequently subjected to in-gel tryptic digestion. The digested peptides were analyzed by LC–MS/MS on Orbitrap Fusion Lumos mass spectrometer (Thermo Scientific, San Jose, CA). Protein identification and relative quantification were performed using Andromeda and MaxQuant (version 1.3.0.5)^51^. The subsequent bioinformatics and statistical analyses were performed with Perseus 1.4.1.3 (http://www.maxquant.org).

### Western blotting

Protein sample was lysed in Novex LDS sample buffer containing DTT reducing agent (Thermo Fisher), heated at 70 °C for 10 min, and then loaded onto Novex NuPAGE 4–12% gradient Bis-Tis gel. Separated proteins were transferred to nitrocellulose membrane and blocked with blocking buffer (5% non-fat milk in TBST (20 mM Tris–HCl, pH 7.4, 110 mM NaCl, 5 mM MgCl2, 0.1% Tween 20). Primary antibodies (Table S13) were prepared in the blocking solution and membranes were incubated overnight at 4 °C. The membrane was washed with TBST and incubated with HRP-conjugated secondary antibodies in TBST for 1 h at room temperature. The membrane was washed and developed with the HRP substrate (WesternBright ECL, Advansta).

### Plasmid construction

To construct human cDNA library, total RNA was extracted from hsSKM cells using RNAiso Plus (Takara) and cDNA was synthesized using TransScript First-Strand cDNA Synthesis SuperMix (TransGen Biotech) in accordance with the manufacturer’s protocol. Using primers listed in Table S12 and the human cDNA library as a template, candidate genes were amplified by PCR and cloned into pcDNA3.6-HA (HA-tag at the C-terminal) vector^13^.

### Immunofluorescence microscopy of the candidate protein tagged with HA

HEK cells were plated on a poly-lysine-coated cover glass, transfected with a plasmid using LipoGeneTM 2000 Star Transfection reagent (US EVERBRIGHT® INC, China), and incubated for 24 h. The cells were fixed with 4% formaldehyde in PBS-D (PBS containing 1 mM of Ca2 + and Mg2 +) for 20 min, rinsed in PBS-D, permeabilized with 0.5% Triton X-100 in TBS (50 mM Tris–HCl, pH 7.4, 150 mM NaCl) for 10min, rinsed in TBS-0.1%Tx (TBS containing 0.1% Triton X-100), blocked in 2% BSA in TBS-0.1%Tx for 1 h, and incubated with primary antibody (anti-HA) for 2 h. After several washing with TBS-0.1%Tx, the cells were incubated with anti-Rabbit IgG-Alexa Fluor Plus 594 antibodies (introvigen) and washed with TBS-0.1%Tx. The nucleus was stained with Hoechst 33,342 (1 μg/ml) for 15min. After washing, the specimen was mounted on slide glass with fluorescence mounting medium (Dako) or directly imaged on a tissue culture dish. Cells were imaged on EVOS® FL Auto Imaging System (Thermo Fisher) fitted with an EVOS® Obj, Inf Plan Fluor 20X LWD objective and Plan Fluor 40X LWD, 0.65NA/2.8WD. Images were processed using Image J software (NIH).

### Direct Immunofluorescence microscopy

Cells were plated on 96-well plates coated with or without 0.1% gelatin (R00503, LEAGENE, China). The cells were fixed as described above and incubated with primary antibodies listed in Table S13 for 2 h. After several washes with TBS-0.1%Tx, the cells were incubated with anti-Rabbit IgG-Alexa Fluor Plus 594 antibodies (introvigen), washed with TBS-0.1%Tx, and directly imaged on a tissue culture dish as described above.

### DSP-MNase-DNA-seq

DNA–protein complexes digested by MNase were treated with proteinase K (CST) to remove bound protein. The DNA was loaded on 1.5% agarose gel, and then purified by E.Z.N.A. R Gel Extraction Kit (Omega BIO-TEK) (around 50 bp band). The library construction was carried out using TruSeq DNA PCR-free prep kit (20,015,963, illumina). Library was sequenced by NovaSeq Sequencer. The fastq data were trimmed with Trim Galore (Galaxy Version 0.6.7), quality checked by FastQC (Galaxy Version 0.73), and mapped to human genome hg38 using Bowtie2 (Galaxy Version 2.4.2). The bigwig files were generated by bamCoverage (Galaxy Version 3.5.1.0.0) and peaks were visualized on integrative genomics viewer (IGV: ver. 2.9.4). Heatmaps for score distributions across genomic regions were created using computeMatrix (Galaxy Version 3.5.1.0.0) and plotHeatmap (Galaxy Version 3.5.1.0.1). To generate narrowPeak files for genomic regions enrichment of annotations (GREAT version 4.0.4: http://great.stanford.edu/public/html/) analysis and annotation analysis using ChIPpeakAnno package (ver. 3.30.1) on RStudio (ver. 2022.02.3 Build 492), macs2 (ver. 2.2.7.1) was run on Mac Terminal (ver. 2.11) to generate a narrowPeak file. To screen for genomic features in A (eg. Low density) that have no overlap in B (eg. High density), bedtools (Version 2.30.0) intersect with “-v” option was used on Mac Terminal. The resulting file was analyzed on GREAT, and genes associated with genomic region was downloaded. The gene name was converted to ENSEMBL gene ID on g:Profiler (https://biit.cs.ut.ee/gprofiler/convert) and analyzed on Functional Annotation tool: DAVID (https://david.ncifcrf.gov/tools.jsp). ChIP-seq data for histone H3 (GEO: GSE213851), H3K4me3 (GEO: GSM733637) and H3K79me2 (GEO: GSM733741) are from public database.

### RNA sequencing

Public RNAseq data for PTGES3 CBFB and TIPARP were obtained from NIH SRA site (https://www.ncbi.nlm.nih.gov/sra). The following analysis were performed on Galaxy (https://usegalaxy.org). The downloaded fastq data were trimmed with Trim Galore (Galaxy Version 0.6.7). The reads were counted by Salmon quant (Galaxy Version 1.5.1), summarized using tximport (Galaxy Version 1.22.0), and converted to csv file on Excel. The RNAseq data were analyzed by integrated differential expression and pathway (iDEP) (http://bioinformatics.sdstate.edu/idep/).

### ChIP-seq motif analysis

The DNA–protein complex was purified as described above. In total, 600 μL of supernatant from six 10 cm dishes (100 μL each) was pooled. The supernantant was incubated with 2 μg rabbit anti-CBF-beta antibody (Cell Signaling Technologies), 5 μg mouse anti-YAP1 antibody (63.7) (Santa Cruz Biotechnologies) or 2 μg rabbit normal IgG (a technical negative control, Cell Signaling Technologies) overnight with rotation, then incubated with 30 μL Protein G Magnetic Beads for 2 h with rotation in accordance with SimpleChIP Enzymatic Chromatin IP kit (Cell Signaling Technologies) with slight modifications as follows. The magnetic beads were pelleted by magnetic separation rack, and then washed 3 times with 1 mL low-salt buffer at 4 °C for 5 min with rotation. The magnetic beads were washed once with 1 mL of high salt buffer at 4 °C for 5 min with rotation and then incubated with 150 μL 1xChIP Elution buffer at 65 °C with vortexing briefly every 2–3 min for 30 min in total. The magnetic beads mixture was incubated with 2 μL Proteinase K (Cell Signaling Technologies) and 10 mM DL-Dithiothreitol (LEAGENE) at 65 °C for 2 h to remove the bound proteins. The eluted DNA fragments were purified using E.Z.N.A. R Cycle-Pure Kit (Omega BIO-TEK) and subjected for library construction.

For ChIP-YAP1-seq analysis, raw reads were trimmed using TrimGalore (Galaxy Version 0.6.7) on Galaxy (https://usegalaxy.org) to remove extra adapters and then mapped to the reference genome (GRCh38 for human) using Bowtie2 (Galaxy Version 2.4.2) to generate bam file. A narrowPeak file was generated by MACS2 callpeak (Galaxy Version 2.2.7.1 + galaxy0) from the bam file. Candidate peaks with *p* values = 0.05 were called. Fasta file was generated by bedtools getfasta (Galaxy Version 2.30.0 + galaxy1) from the narrowPeak file. Motif analysis was performed by MEME-ChIP (version 5.5.2) (https://meme-suite.org/meme/tools/meme-chip) using HOCOMOCO Human (v11 CORE) as input motifs (https://meme-suite.org/meme/tools/meme-chip). For CBFB, ChIP-seq analysis was performed as above except for using p-values = 0.0005 on MACS2.

### Measurement of the subcellular protein localization in cells

Fluorescent intensity along a line (examples in Figure S3) in an immunofluorescent image (with the blue channel representing the nucleus and the red channel representing the protein of interest) was quantified using the "Plot Profile" tool in NIH Image J (version 2.9.0/1.53t). The Pearson's correlation coefficient, which varies from − 1 (indicating perfect cytosolic localization) to 1 (indicating perfect nuclear localization), was computed using GraphPad Prism 9.0.0 to assess the correlation between the intensities of the blue and red channels.

### Statistics

All experiments were independently performed at least three times, except for proteomic and genomic analyses. Significance was assessed using unpaired t-test with GraphPad Prism 9.0.0. *P < 0.05, **P < 0.01, ***P < 0.001, ****P < 0.0001. All image analysis was performed by operators who were blinded to the treatments administered.

## Data and code availability

The data supporting the findings of this study are available within the article and its supplementary files. All original data are available from the corresponding author upon reasonable request. Deep-sequencing data in this study have been deposited in the Gene Expression Omnibus (GEO) (DSP-MNase-DNA-seq: GSE221380, ChIP-seq: GSE234731). The data were released to the public on December 5th, 2023.

### Supplementary Information

Supplementary Information 1.

Supplementary Information 2.

## References

[1] Roycroft A, Mayor R (2018). Michael Abercrombie: contact inhibition of locomotion and more. Int. J. Dev. Biol..

[2] Stramer B, Mayor R (2017). Mechanisms and in vivo functions of contact inhibition of locomotion. Nat. Rev. Mol. Cell Biol..

[3] Dupont S (2011). Role of YAP/TAZ in mechanotransduction. Nature.

[4] Pavel M (2018). Contact inhibition controls cell survival and proliferation via YAP/TAZ-autophagy axis. Nat. Commun..

[5] Ahmad K, Henikoff S, Ramachandran S (2022). Managing the steady state chromatin landscape by nucleosome dynamics. Annu. Rev. Biochem..

[6] Chen YC, Koutelou E, Dent SYR (2022). Now open: Evolving insights to the roles of lysine acetylation in chromatin organization and function. Mol. Cell.

[7] Aoki T (2014). Bi-functional cross-linking reagents efficiently capture protein-DNA complexes in Drosophila embryos. Fly Austin.

[8] Cox J (2009). A practical guide to the MaxQuant computational platform for SILAC-based quantitative proteomics. Nat. Protoc..

[9] Fang Y (2016). Functional characterization of open chromatin in bidirectional promoters of rice. Sci. Rep..

[10] Kong S (2022). Nucleosome-omics: A perspective on the epigenetic code and 3D genome landscape. Genes Basel.

[11] Mansisidor AR, Risca VI (2022). Chromatin accessibility: methods, mechanisms, and biological insights. Nucleus.

[12] Gumbiner BM, Kim NG (2014). The Hippo-YAP signaling pathway and contact inhibition of growth. J. Cell Sci..

[13] Feng M, Wang J, Li K, Nakamura F (2023). UBE2A/B is the trans-acting factor mediating mechanotransduction and contact inhibition. Biochem. J..

[14] Deng WH, Li XH (2020). Resolving nucleosomal positioning and occupancy with MNase-seq. Yi Chuan.

[15] Klein DC, Hainer SJ (2020). Genomic methods in profiling DNA accessibility and factor localization. Chromosome Res..

[16] Wu J (2020). Advances in assay for transposase-accessible chromatin with high-throughput sequencing. Yi Chuan.

[17] Kondili M (2017). UROPA: A tool for universal robust peak annotation. Sci. Rep..

[18] McLean CY (2010). GREAT improves functional interpretation of cis-regulatory regions. Nat. Biotechnol..

[19] Sherman BT (2022). DAVID: A web server for functional enrichment analysis and functional annotation of gene lists (2021 update). Nucleic Acids Res..

[20] Aragona M (2013). A mechanical checkpoint controls multicellular growth through YAP/TAZ regulation by actin-processing factors. Cell.

[21] Yue P (2022). Yap1 modulates cardiomyocyte hypertrophy via impaired mitochondrial biogenesis in response to chronic mechanical stress overload. Theranostics.

[22] Coto-Llerena M (2021). Transcriptional enhancer factor domain family member 4 exerts an oncogenic role in hepatocellular carcinoma by hippo-independent regulation of heat shock protein 70 family members. Hepatol. Commun..

[23] Yamashiro T, Kurosaka H, Inubush T (2022). The association between runx signaling and craniofacial development and disease. Curr. Osteoporos. Rep..

[24] Shin MH (2016). A RUNX2-mediated epigenetic regulation of the survival of p53 defective cancer cells. PLoS Genet..

[25] Dutta B, Yan R, Lim SK, Tam JP, Sze SK (2014). Quantitative profiling of chromatome dynamics reveals a novel role for HP1BP3 in hypoxia-induced oncogenesis. Mol. Cell Proteomics.

[26] Federation AJ (2020). Highly parallel quantification and compartment localization of transcription factors and nuclear proteins. Cell Rep..

[27] Kulej K (2017). Time-resolved global and chromatin proteomics during herpes simplex virus type 1 (HSV-1) Infection. Mol. Cell Proteomics.

[28] Garcia-Garcia M (2022). Mechanical control of nuclear import by Importin-7 is regulated by its dominant cargo YAP. Nat. Commun..

[29] Giresi PG, Lieb JD (2009). Isolation of active regulatory elements from eukaryotic chromatin using FAIRE (formaldehyde assisted isolation of regulatory elements). Methods.

[30] Hoffman EA, Frey BL, Smith LM, Auble DT (2015). Formaldehyde crosslinking: A tool for the study of chromatin complexes. J. Biol. Chem..

[31] MacPherson L (2013). 2,3,7,8-Tetrachlorodibenzo-p-dioxin poly(ADP-ribose) polymerase (TiPARP, ARTD14) is a mono-ADP-ribosyltransferase and repressor of aryl hydrocarbon receptor transactivation. Nucleic Acids Res..

[32] Bock KW (2021). Aryl hydrocarbon receptor (AHR) functions in infectious and sterile inflammation and NAD(+)-dependent metabolic adaptation. Arch. Toxicol..

[33] Andrysik Z (2013). Aryl hydrocarbon receptor-mediated disruption of contact inhibition is associated with connexin43 downregulation and inhibition of gap junctional intercellular communication. Arch Toxicol..

[34] Li Y (2022). Growth of T-cell lymphoma cells is inhibited by mPGES-1/PGE2 suppression via JAK/STAT, TGF-beta/Smad3 and PI3K/AKT signal pathways. Transl Cancer Res..

[35] McCarthy AJ, Chetty R (2018). Smad4/DPC4. J. Clin. Pathol..

[36] Hamaratoglu F (2006). The tumour-suppressor genes NF2/Merlin and expanded act through Hippo signalling to regulate cell proliferation and apoptosis. Nat. Cell Biol..

[37] Elosegui-Artola A (2017). Force triggers YAP nuclear entry by regulating transport across nuclear pores. Cell.

[38] Morgunova E, Taipale J (2021). Structural insights into the interaction between transcription factors and the nucleosome. Curr. Opin. Struct. Biol..

[39] Tsunaka Y, Furukawa A, Nishimura Y (2022). Histone tail network and modulation in a nucleosome. Curr. Opin. Struct. Biol..

[40] Bernstein BE (2002). Methylation of histone H3 Lys 4 in coding regions of active genes. Proc. Natl. Acad. Sci. U. S. A..

[41] Martin C, Zhang Y (2005). The diverse functions of histone lysine methylation. Nat. Rev. Mol. Cell Biol..

[42] Kim TH (2005). A high-resolution map of active promoters in the human genome. Nature.

[43] Berger SL (2007). The complex language of chromatin regulation during transcription. Nature.

[44] Barski A (2007). High-resolution profiling of histone methylations in the human genome. Cell.

[45] Robert F, Jeronimo C (2023). Transcription-coupled nucleosome assembly. Trends Biochem. Sci..

[46] Sekine SI, Ehara H, Kujirai T, Kurumizaka H (2023). Structural perspectives on transcription in chromatin. Trends Cell Biol..

[47] Furlong EEM, Levine M (2018). Developmental enhancers and chromosome topology. Science.

[48] Maeshima K, Iida S, Shimazoe MA, Tamura S, Ide S (2023). Is euchromatin really open in the cell?. Trends Cell Biol..

[49] Wang L, Nakamura F (2019). Identification of filamin A mechanobinding partner I: Smoothelin specifically Interacts with the Filamin A mechanosensitive domain 21. Biochemistry.

[50] Sun Y, Sriramajayam K, Luo D, Liao DJ (2012). A quick, cost-free method of purification of DNA fragments from agarose gel. J. Cancer.

[51] Cox J (2011). Andromeda: a peptide search engine integrated into the MaxQuant environment. J. Proteome Res..

