# Supplementary material for: Identification and partial characterization of new cell density-dependent nucleocytoplasmic shuttling proteins and open chromatin
Source: Sci Rep. 2023 Dec 8;13:21723. doi: 10.1038/s41598-023-49100-6 (PMC10709462; doi:10.1038/s41598-023-49100-6)
Supplement: Supplementary file 1 — Supplementary Information 1. [file 41598_2023_49100_MOESM1_ESM.pdf]

Supplementary information

Identification and partial characterization of new cell density-dependent nucleocytoplasmic shuttling proteins and open chromatin

Kangjing Li, Yaxin Li, Fumihiko Nakamuura

Correspondence to: fnakamura@tju.edu.cn

s

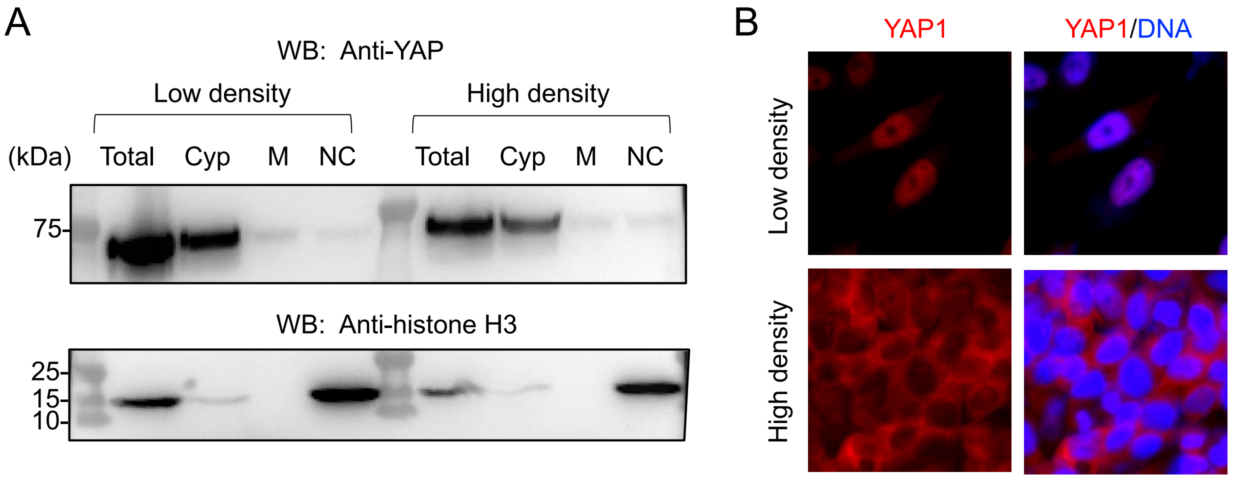

Figure S1. Commercial fractionation kit does not retain YAP1 in "nuclear fraction". (**A**) hsSKM cells were cultured at low and high density and fractionated using Cell Signaling Technology cell fractional kit (#9038) (Cyp: Cytoplasmic, M: Membrane, NC: Nucleus and cytoskeleton). YAP1 and histone H3 were detected by western blotting using mouse anti-YAP and histone H3 antibodies, respectively. (**B**) Immunofluorescence of hsSKM cells at low and high density. YAP1 was stained with anti-YAP1 antibody followed by Alexa 594 secondary antibody. Nuclei were stained with Hoechst 33342. Scale: 100 × 100 μm.

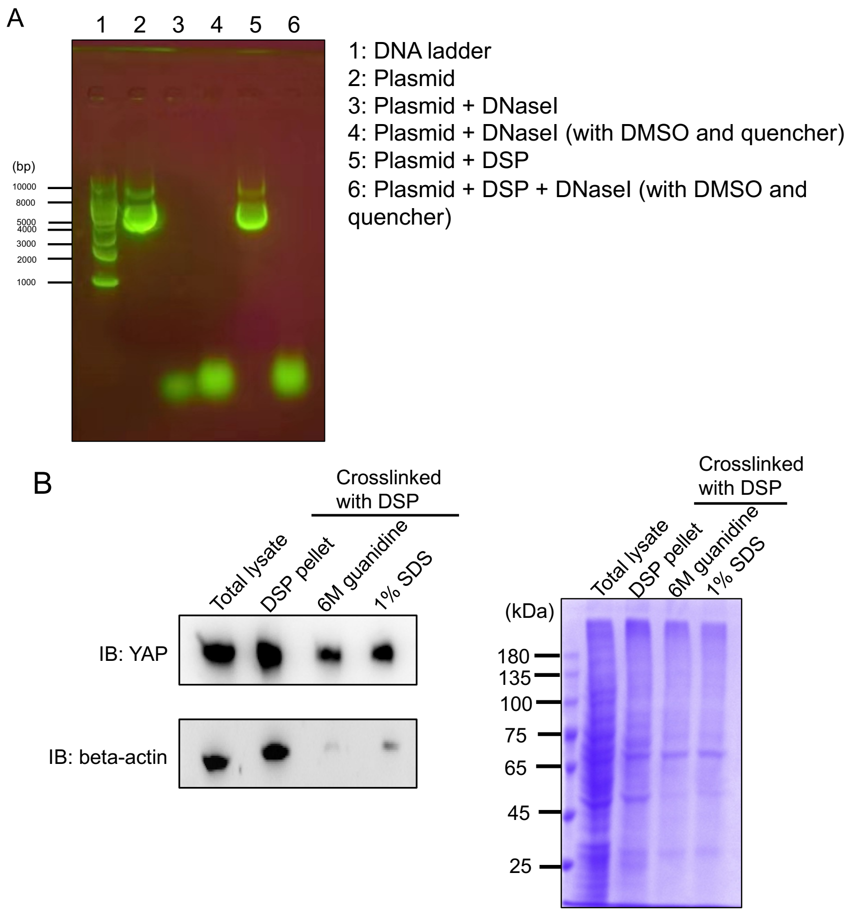

Figure S2. Reactivity of DSP to DNA and enrichment of protein-DNA complex by washing. (**A**) pcDNA3-ZBTB2-HA (1 μg) diluted in water with primary amine was digested with 1 μl of DNase I (1U/μl) in 10 μl of PBS for 10 min at 37℃. For same experiment, plasmid was incubated with 2.5 μl of 5mM DSP solubilized in DMSO (final concentration is 1mM) for 30 min at room temperature. The reaction was stopped by adding 0.375 μl of 1M Tris-HCl, pH8.0. Note that protein-free plasmid DNA is non-reactive to DSP. DNA was analyzed on 0.8% agarose gel and stained with SuperGreen (Biosharp). (**B**) Total lysate is the cells lysed with RIPA-T buffer. DSP pellet is the pellet fraction from cells that were crosslinked with DSP, lysed with RIPA-T buffer, and then centrifuged. The cells were also lysed with RIPA-T buffer containing 6M guanidine (RIPA-G) or 1% SDS. The pellets were washed with PBS several times to remove remaining detergents. Note that additions of these detergents remove cytoskeletal actin and majority of other proteins (right panel: CBB stained SDS-PAGE gel), while retaining YAP in the pellet. Since we noticed that addition of 1% SDS reduces the efficiency of the following MNase digestion, we use RIPA-G in our experiment.

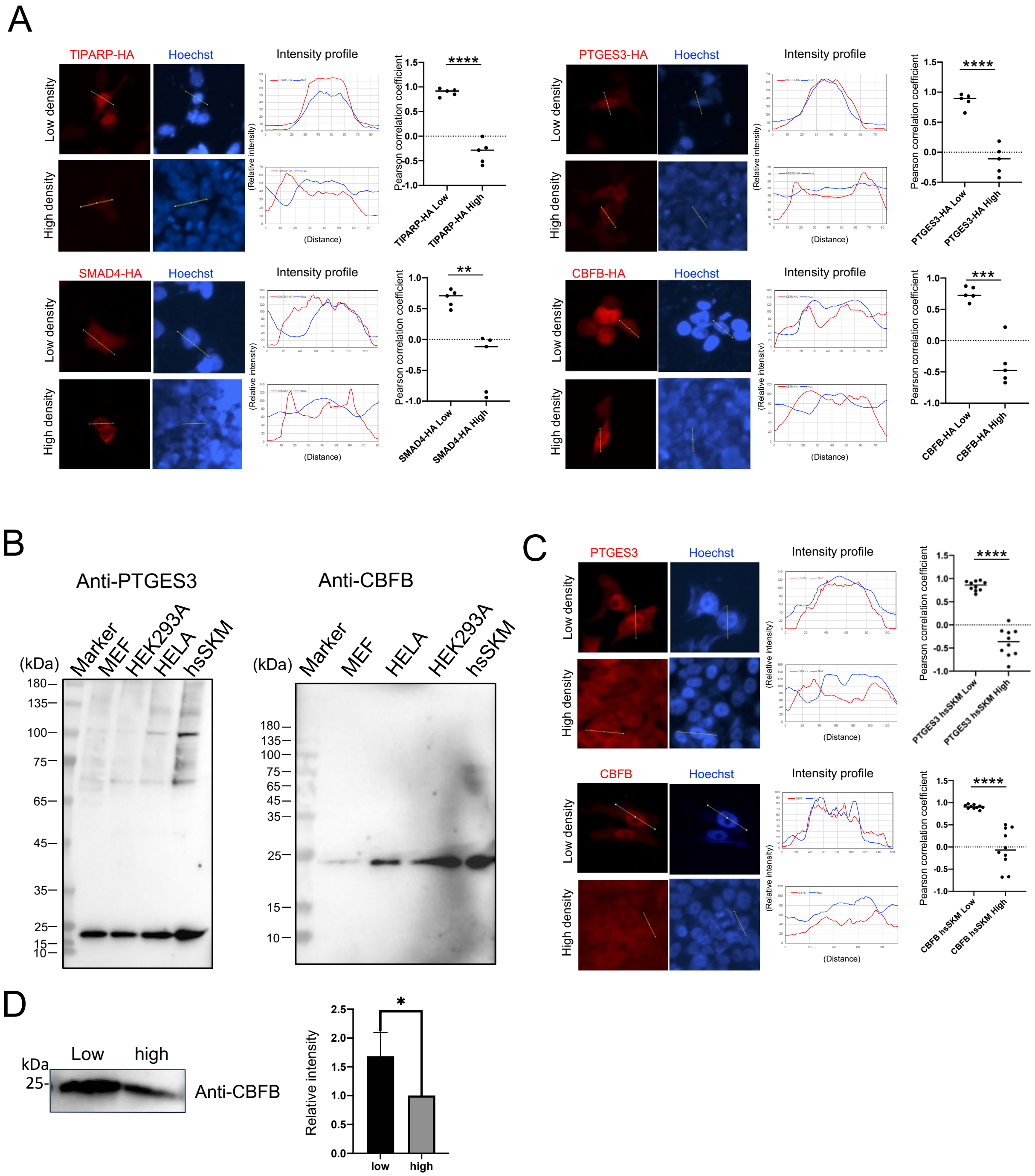

Figure S3. Cell density dependent translocation of TIPARP, PTGES3, SMAD4, and CBFB.

(**A**) HEK 293 cells were cultured at either low or high density and transfected with either pcDNA3-TIPARP-HA, pcDNA3-PTGES3-HA, or pcDNA3-SMAD4-HA. For CBFB, hsSKM cells were transfected with pcDNA3-CBFB-HA. Expressed proteins were detected by immunofluorescent microscopy using anti-HA antibody following by secondary antibody conjugated with Alexa594. (**B**) Western blotting showing specificity of anti-PTGES3 and anti-

CBFB antibodies used for immunofluorescent microscopy. Mouse embryonic fibroblast (MEF). (**C**) HEK 293 cells were cultured at either low or high density for the detection of PTGES3, and hsSKM cells were cultured at either low or high density for the detection of CBFB. Both cell types were then fixed. PTGES3 was detected using immunofluorescent microscopy with a specific anti-PTGES3 antibody, followed by a secondary antibody conjugated with Alexa594. CBFB was detected using immunofluorescence with a specific anti- PEBPβ (CBFB) mouse monoclonal antibody, followed by a secondary antibody conjugated with Alexa594. Nuclei were stained with Hoechst 33342. Scale: 100 $\times$ 100 μm. Fluorescent intensity on yellow line was measured by "Plot Profile" on NIH Image J using. Red line indicates the intensity of staining of a protein of interest. Blue line indicates the intensity of nuclear staining. Pearson's correlation coefficient, calculated with GraphPad Prism 9.0.0, was used to evaluate the correlation between the intensities of the blue and red channels and then plotted. Pearson's correlation coefficient ranges from -1 (representing perfect cytosolic localization) to 1 (representing perfect nuclear localization). Each data point represents a measured cell (n=5~10). Significance was analyzed by unpaired t-test. *P<0.05, **P<0.01, ***P<0.001, ****P<0.0001. ns: not significant. (**D**) Biochemical fractionation of CBFB. The hsSKM cells cultured at low and high density were crosslinked with DSP, and the DNA-protein complex was extracted following the procedure detailed in Figure 3. The total protein quantity was standardized using western blotting against histone before centrifugation. The CBFB quantity in each fraction was determined through western blotting, and the relative band intensity was plotted. Significance was analyzed by unpaired t-test. *P<0.05 (n=3). Due to the ineffectiveness of the anti-PTGES3 antibody used for immunofluorescent microscopy in western blotting after cross-linking with DSP, the fractionation of PTGES3 couldn't be tested.

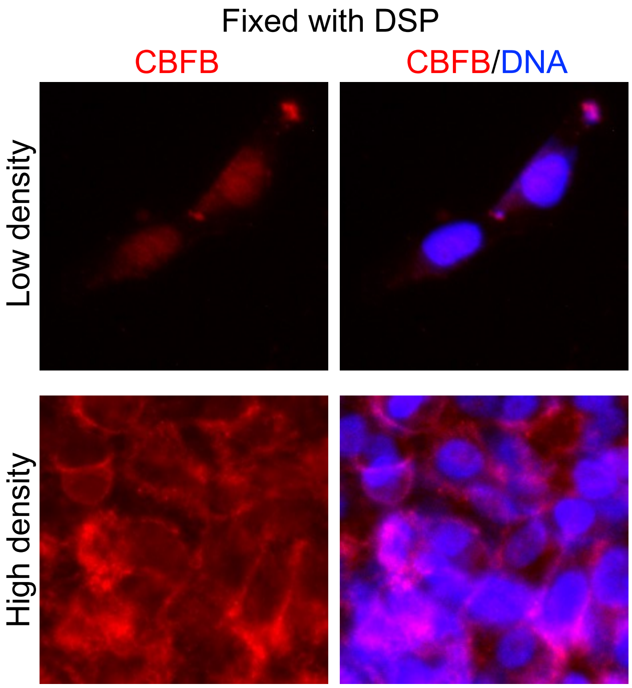

Figure S4. DSP crosslinking does not interfere staining of CBFB by immunofluorescent microscopy.
hsSKM cells were fixed with DSP and CBFB was detected by immunofluorescent using anti-CBFB rabbit polyclonal antibody following by secondary antibody conjugated with Alexa594. Similar result was obtained with anti-CBFB rabbit monoclonal antibody (CST) used for ChIP-seq. Nuclei were stained with Hoechst 33342. Scale: 100 × 100 μm.

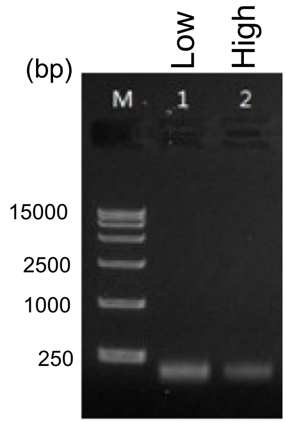

Figure S5. DNA sample for NGS analysis.
After MNase digestion, soluble DNA-protein complex was separated on agarose gel and the complex was extracted from the gel. Bound protein was removed by de-crosslinking and proteinase K digestion and DNA was purified using spin column. Note that purified DNA migrated around 200 bp.

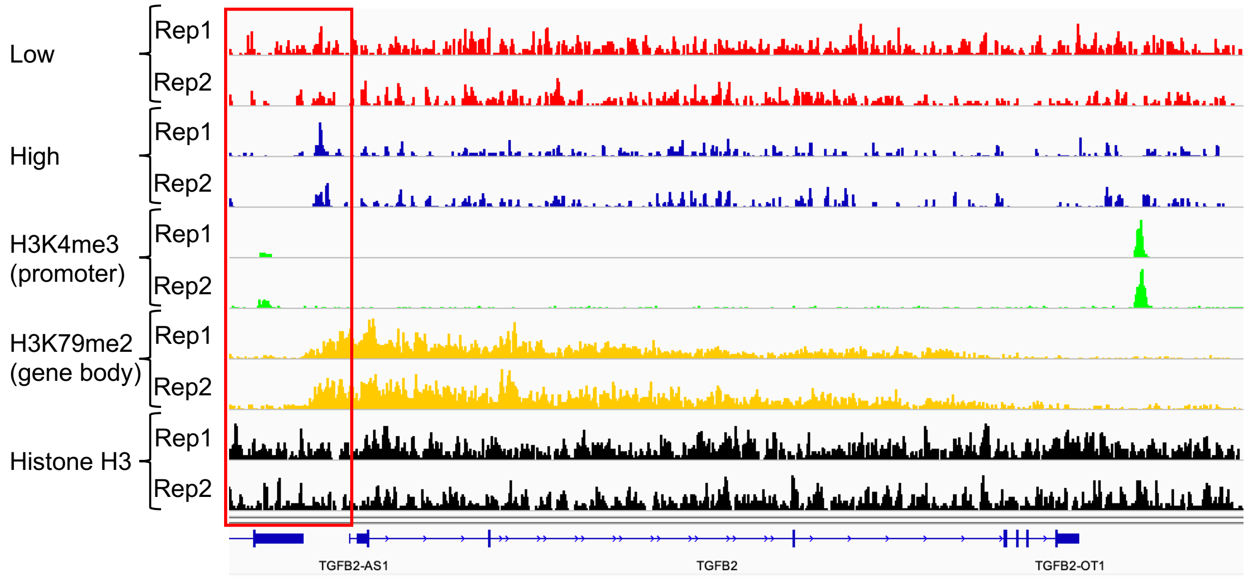

Figure S6. MNase-sensitive sites on chromatin and the histone modification landscape in human skeletal muscle cells.
Representative integrative genomics view of MNase-sensitive sites on chromatin purified from low- and high-density cells, and the H3K4me3 density (GSM733637), the H3K79me2 density (GSM733741), and the histone H3 density (GSE213851) at the TGFB2 region in human skeletal muscle cells. Red square indicates upstream of TGFB2 which is a target of CBFB.

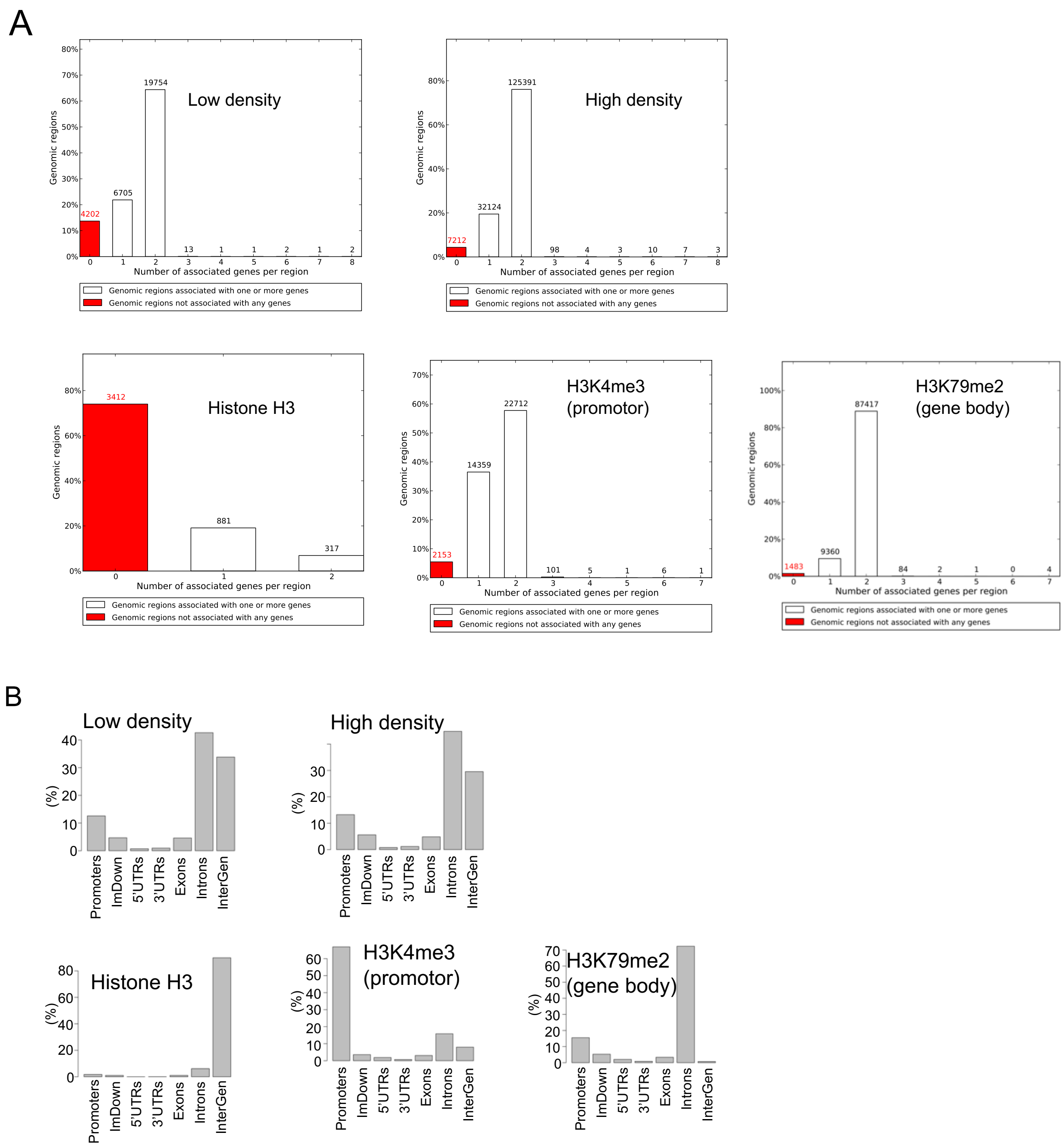

Figure S7. Annotation of MNase-digested DNA fragments from DSP-crosslinked cells by GREAT analysis.

(**A**) MNase-digested DNA fragments from cells at both low and high densities were subjected to NGS, and bed files were generated using the Galaxy platform (https://usegalaxy.org). For comparative purposes, publicly available ChIP-seq data for histone H3, H3K4me3, and H3K79me2 were utilized. Sequencing reads were aligned using Bowtie2, and macs2 was employed to create the narrowPeak file. After modifying the narrowPeak file to ensure compatibility with GREAT analysis, the data was assessed via GREAT

(http://great.stanford.edu/public/html/) to identify the genes associated with these genomic regions. In the graphical representation, white bars denote genomic regions linked to one or more genes, while red bars signify genomic regions not associated with any genes. Notably, a significant proportion of peaks in the histone H3 ChIP-seq data are unrelated to protein-coding genes. In contrast, MNase-digested DNA fragments from both low- and high-density cells exhibit associations with multiple genes, similar to H3K4me3 and H3K79me2. (**B**) Regional distribution. From left to right, Prom: Promoters, Immediate Downstream (ImDown), 5'UTRs, 3'UTRs, Exons, Introns, and intergenic region (InterGen: non-protein-coding). Notably, the majority of the peaks in histone H3 ChIP-seq are mapped to the intergenic non-protein-coding region. However, similar to H3K4me3 ChIP-seq data, approximately 13% of MNase-digested DNA fragments from both low (12.6 %) and high (13.2 %) density cells are mapped to promoter region (H3K4me3: 14.3 %).

Source image data for Figure 1, Figure 3ABC, Figure S1A, Figure S2AB, Figure S5

Only the lanes outlined in red pertain to this paper.

Figure 1.

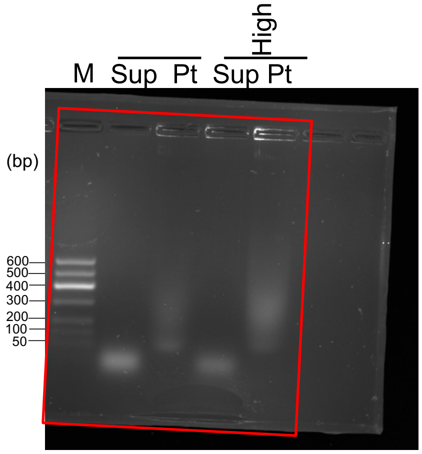

Figure 3A

1: Control

2: pellet

3: sup

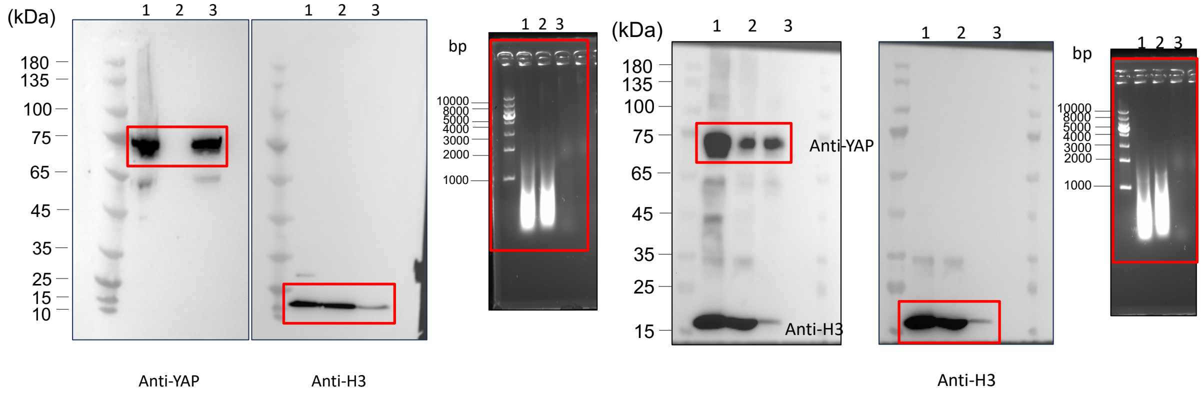

Figure 3B

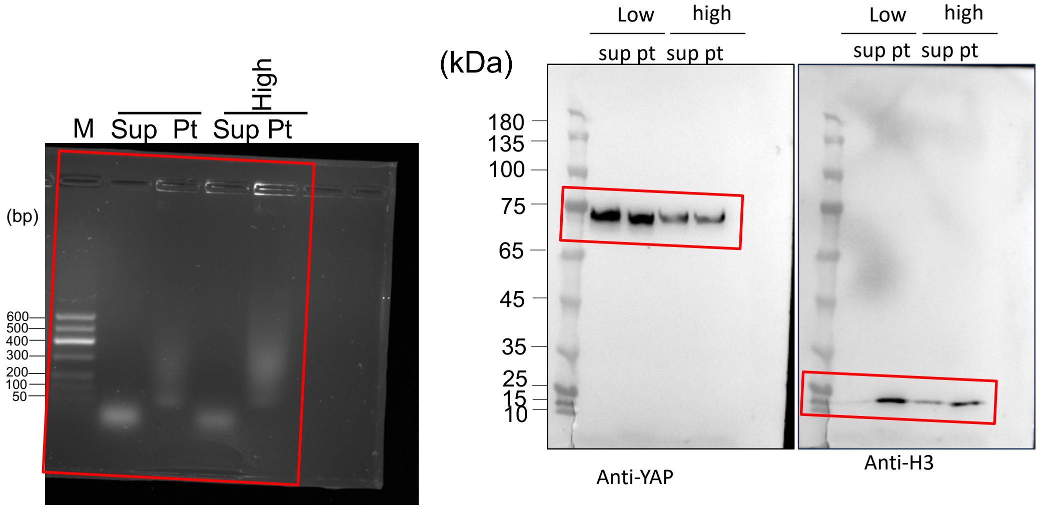

Figure 3C

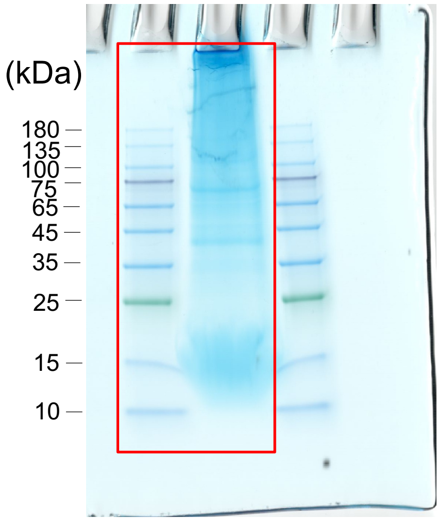

Figure S1A

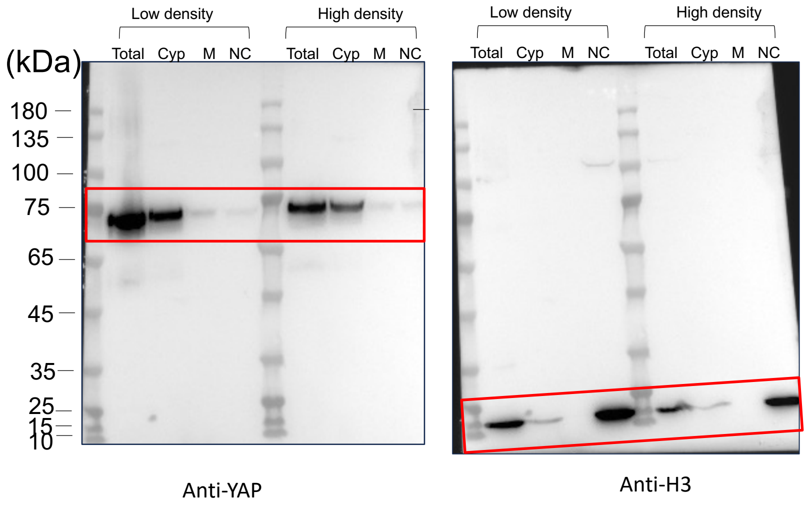

Figure S2

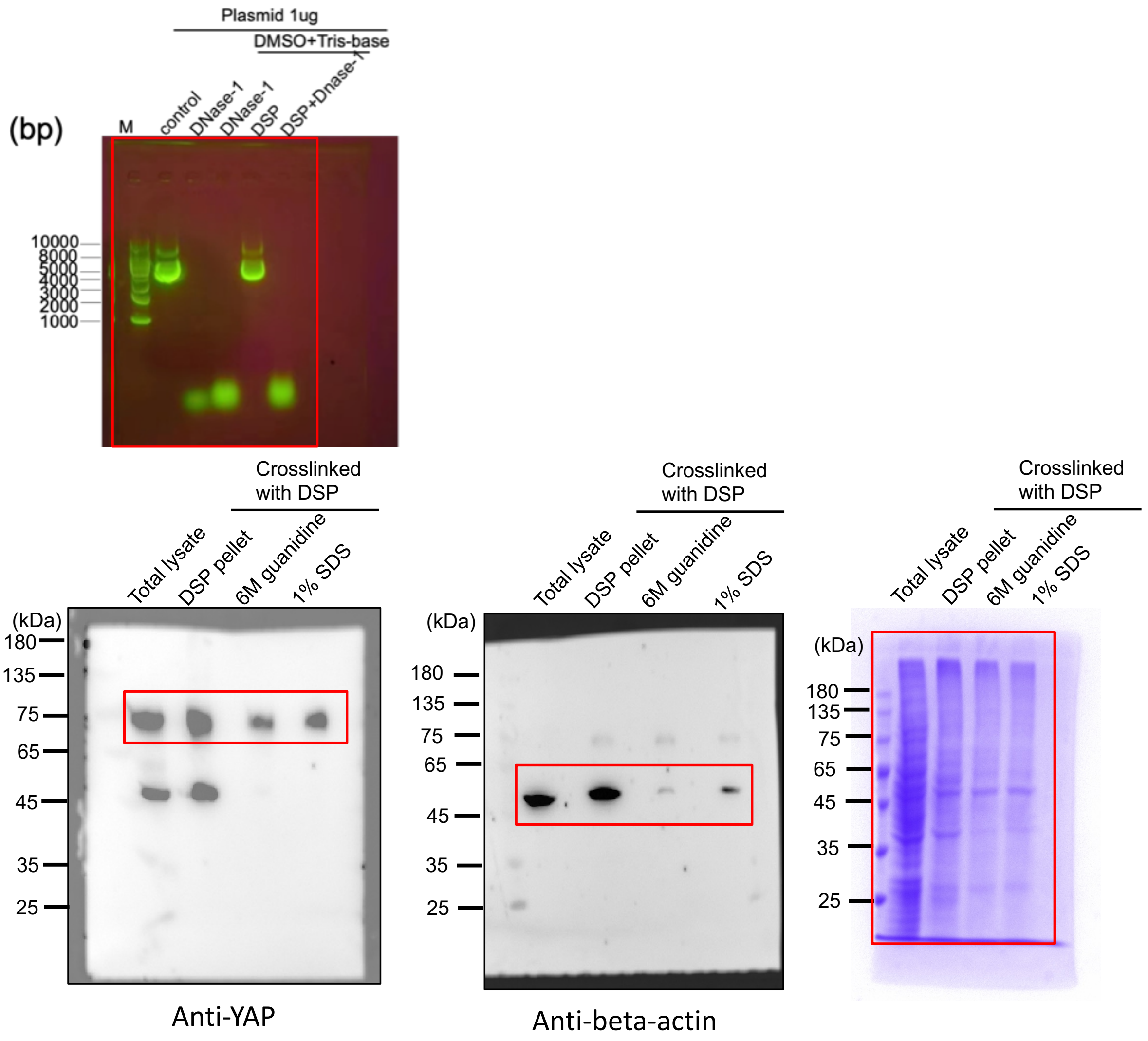

Figure S3

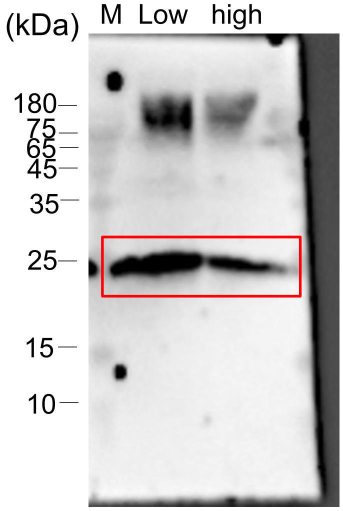

Figure S5

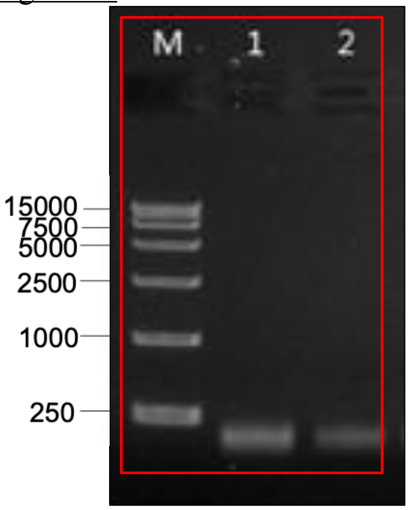

1. Low density 2. High density

Table S1. List of identified peptides by conventional mass spectrometry.

Proteins isolated from the DNA-protein complex of low-density cells were digested with trypsin and subjected to mass spectrometry.

Table S2. Transcription related proteins identified by the conventional mass spectrometry.

Proteins detected by conventional mass spectrometry (as shown in Table S1) were searched using the UniProt database to screen for only transcription-related proteins.

Table S3. List of identified peptides by SILAC-based mass spectrometry.

hsSKM cells cultured at low density were labeled with heavy amino acids, while hsSKM cells cultured at high density were labeled with light amino acids, as shown in Figure 1. The proteins isolated from the DNA-protein complex were separated on a NuPAGE gel, cut into 9 fragments (as depicted in Figure 3C), digested with trypsin, and then subjected to mass spectrometry. Because of shift of the data points of actin (ACTB and ACTG1) and tubulin (TUBA) peptides to left side on the scatterplot, H/L ratio was re-normalized as shown in column BE~BG.

Table S4. List of proteins with positive Log_2(H/L).

Proteins whose peptides have positive H/L ratio and H/L ratio with NaN were screened. Peptides from 1220 different proteins were mainly detected on the right (positive Log(H/L)) side of the scatterplot, indicating that these 1220 proteins were enriched in low density cells.

Table S5. List of potential mechanosensitive proteins.

The 52 candidate proteins displayed in Table S2 column B, and the 179 proteins shown in Table S4 column B were further evaluated using immunofluorescence images on the Human Protein Atlas (https://www.proteinatlas.org/). Proteins that were detected in the nucleus, cytosol, or both, as determined by immunofluorescence microscopy, were listed. It is noteworthy that 17 potential mechanosensitive proteins were identified. Normalized H/L ratio of detected peptides and NaN=Not a Number are also indicated.

Table S6. RNA-seq expression data for PTGES3 knock-out (KO) human hepatoma HepG2 cells.

Publicly available RNA sequencing data (BioProject: PRJNA30709) was analyzed using Salmon quant (Galaxy Version 1.5.1). The data can be further analyzed on iDEP (http://bioinformatics.sdstate.edu/idep/).

Table S7. RNA-seq expression data for PTGES3 KO human immortalized myelogenous leukemic K562 cells.

Publicly available RNA sequencing data (BioProject: PRJNA30709) was analyzed using Salmon quant (Galaxy Version 1.5.1). The data can be further analyzed on iDEP (http://bioinformatics.sdstate.edu/idep/).

Table S8. RNA-seq expression data for TIPARP/PARP7 knock-down (KD) human ovarian carcinoma OVCAR4 cells.

Publicly available RNA sequencing data (BioProject: PRJNA642215) was analyzed using Salmon quant (Galaxy Version 1.5.1). The data can be further analyzed on iDEP (http://bioinformatics.sdstate.edu/idep/).

Table S9. RNA-seq expression data for CBFB KO human breast epithelial MCF10A cells.

Publicly available RNA sequencing data (BioProject: PRJNA492137) was analyzed using Salmon quant (Galaxy Version 1.5.1). The data can be further analyzed on iDEP (http://bioinformatics.sdstate.edu/idep/).

Table S10. Genes associated with the DNA sequences detected by the DSP-MNase proteogenomics.

Genes associated with the DNA sequences detected by the DSP-MNase proteogenomics were analyzed using the GREAT database (http://great.stanford.edu/public/html/). The genes are represented in the Gene Name format.

Table S11. Genes associated with the DNA sequences detected by the DSP-MNase proteogenomics.

Genes associated with the DNA sequences detected by the DSP-MNase proteogenomics were analyzed using the GREAT database (http://great.stanford.edu/public/html/). The genes are represented in the GENCODE ID format.

Table S12. Primers used to construct pcDNA3.6-HA vector.

Using primers listed in Table S12 and the human cDNA library as a template, candidate genes were amplified by PCR and cloned into pcDNA3.6-HA vector.

Table S13. Antibodies used for Immunofluorescence microscopy, Chip-seq, and WB.

Commercial antibodies used in this project.
